# Understanding barriers to stool adequacy: results from a programmatic assessment of Pakistan's acute flaccid paralysis active surveillance system in 12 priority districts

**DOI:** 10.3389/fpubh.2025.1549291

**Published:** 2025-05-09

**Authors:** Keri Geiger, Abul Aziz, Chukwuma Mbaeyi, Zainul Abedin Khan, Mohammed Soghaier, Aimee Summers

**Affiliations:** ^1^Centers for Disease Control and Prevention, Atlanta, Georgia; ^2^World Health Organization, Islamabad, Pakistan

**Keywords:** poliovirus, polio eradication, vaccine preventable disease surveillance, active surveillance, surveillance system

## Abstract

**Introduction:**

Pakistan's acute flaccid paralysis (AFP) surveillance system is an essential part of efforts to eradicate poliomyelitis, as Pakistan and Afghanistan are the only countries where wild poliovirus remains endemic. The two primary performance indicators for AFP surveillance are the non-polio AFP rate for children aged <15 years and stool adequacy, defined as the percentage of AFP cases for which two timely stool samples arrive at the laboratory in good condition. Despite consistently meeting targets for both indicators at the national level, some districts in Pakistan failed to meet the stool adequacy target of ≥80% in 2023 or had declining stool adequacy. In March 2024, we assessed AFP surveillance in 12 districts in Pakistan with low stool adequacy to characterize barriers to meeting the target.

**Methods:**

The assessment included review of case investigation forms from AFP cases with patient paralysis onset during January 2023–mid-March 2024 with inadequate stool samples, as well as visits to health facilities serving as active surveillance sites and interviews with surveillance and laboratory personnel.

**Results:**

The most common barrier to stool adequacy was a delay between onset of paralysis and AFP case notification, which occurred in 111 of 158 (70%) inadequately sampled AFP cases reviewed. This delay was most frequently attributed to missed reporting by healthcare facilities, caretakers seeking healthcare many days after paralysis onset, or a combination of both. Additionally, only 63% of health facilities showed adequate active surveillance visit compliance.

**Discussion:**

The assessment exposed gaps in AFP surveillance knowledge for some health facility staff, especially nurses and other paramedical or support professionals. Recommendations to improve the AFP surveillance system include monitoring and encouraging compliance with systematically scheduled health facility visits, increasing the frequency of AFP surveillance orientations, including paramedical professionals in AFP surveillance training, and developing a comprehensive messaging plan to increase knowledge about prompt reporting of AFP among healthcare providers and the public.

## 1 Introduction

Following global efforts and investments since 1988 when the goal of poliomyelitis eradication was declared, wild poliovirus transmission remains endemic only in Pakistan and Afghanistan (1). Poliovirus eradication efforts rely on a sensitive case-based syndromic surveillance system that ensures identification and testing for poliovirus in every case of acute flaccid paralysis (AFP) in children under 15 years of age (2). Substantial investments in Pakistan's AFP surveillance system over many years by the Pakistani government and the Global Polio Eradication Initiative have resulted in a large, complex yet robust and highly functional surveillance system (3). AFP surveillance in Pakistan includes active surveillance, in which polio surveillance staff periodically visit selected healthcare facilities designated as active surveillance sites. During these visits, surveillance staff search for potential AFP cases in facility patient registries and in patient care areas, sign reviewed registries to document their work, and conduct on-the-spot training and sensitization of health staff to promptly report AFP cases. The AFP surveillance system also includes passive surveillance, in which any healthcare provider or community informant can report AFP cases to the district health office (4).

The two most important indicators of AFP surveillance system performance nationally and sub-nationally are the non-polio AFP rate (the number of reported AFP cases determined not to be due to poliovirus infection for a time period divided by population size) and the percentage of stool adequacy among all investigated AFP cases. A high non-polio AFP rate indicates that the surveillance system would likely detect cases of actual poliomyelitis. The target non-polio AFP rate is ≥3 cases per 100,000 children <15 years of age in countries with endemic circulation, compared with ≥2 cases per 100,000 in other outbreak and high-risk settings, and ≥1 case per 100,000 in countries in polio-free regions (1, 3). Because several diseases besides poliovirus infection can cause AFP in children <15 years of age, every AFP case-patient must have stool samples tested for poliovirus to confirm or exclude the diagnosis of poliomyelitis. To reduce the possibility of not confirming a true polio case, ≥80% of AFP case-patients should have adequate stool samples, defined as two stool samples of sufficient quantity for testing, collected at least 24 h apart and within 14 days of paralysis onset (timeliness), and arriving to an accredited poliovirus laboratory in good condition (quality) (2, 4). A stool adequacy percentage ≥80% increases the probability of isolating poliovirus from patients with AFP if the paralysis is caused by poliomyelitis and provides evidence of the absence of poliovirus transmission in the population if the stool results are negative for poliomyelitis.

Because <1% of individuals infected with poliovirus will develop AFP, environmental surveillance, in which wastewater samples systematically obtained at designated sites are tested for poliovirus, supplements AFP surveillance in Pakistan (2). In 2023, the Pakistan National Emergency Operations Center (NEOC) for Polio Eradication reported six wild poliovirus type 1 (WPV1) cases detected through AFP surveillance: four from Khyber Pakhtunkhwa province (three in Bannu district and one in Orakzai district) and two from Sindh province (Karachi city). However, environmental surveillance detected WPV1 in 126 samples from 52 sites located in five provinces (Islamabad, Balochistan, Khyber Pakhtunkhwa, Punjab, and Sindh). During January 1–March 31, 2024, two WPV1 cases were reported from Balochistan province, while environmental surveillance detected WPV1 in 96 samples collected at 47 sites in the same five provinces. The wider geographic spread of WPV1 indicated by environmental detections in provinces and districts with no WPV1 AFP cases led to renewed questions about the performance of Pakistan's AFP surveillance system. The surveillance system is routinely evaluated and consistently meets the target non-polio AFP rate at the national, provincial, and district levels (1, 5). However, some districts showed declining proportion of cases with adequate samples during 2019–2023 or failed to meet stool adequacy targets in 2023 (1, 5). A decline in stool adequacy percentages could result in some poliomyelitis cases testing negative for poliovirus and could partially explain why poliovirus circulation was detected in some areas only through environmental surveillance.

Improving stool adequacy requires identifying barriers affecting either the timeliness of stool sample collection or the quality of the stool sample on arrival to the laboratory. Possible barriers affecting timeliness include delays in caretaker seeking healthcare, late or failed reporting of AFP cases by healthcare facilities and providers, or delays conducting AFP case investigations or obtaining stool samples. Deficiencies in conducting active surveillance health facility visits could contribute to these barriers, specifically missed AFP reporting or delays in case investigation and stool sample collection. Inadequate knowledge or physical barriers leading to inappropriate collection, storage, and transport of stool samples could affect sample quality. Once collected, stool samples must be stored and transported to the laboratory under cold chain conditions. Samples reaching a temperature >8°Centigrade before being processed in the laboratory do not meet sample quality criteria. An additional sample is then obtained from the AFP case-patient. Obtaining an additional sample results in inadequate stool samples if the resample is obtained more than 14 days after paralysis onset (6). In this case, a stool sample should be obtained from three contacts of the AFP case-patient, though the case-patient is still considered to have inadequate stool samples (6). In 2023, Pakistan revised standard operating procedures for shipping stool samples, requiring LogTag™ (LogTag North America, Inc., Lafayette, NJ) devices which record temperature over time, to be included in shipping containers (7). This change led to questions over whether the number of stool samples that arrive at the laboratory with a recorded cold chain breach has increased, causing them to not meet the quality criteria, and whether that increase has caused a decrease in the proportion of AFP cases with adequate stool samples.

Given the potential of missed WPV1 by gaps in AFP surveillance, Pakistan's national polio program requested an assessment of the AFP surveillance system. This assessment focused on stool adequacy because non-polio AFP rates consistently exceeded expected targets while stool adequacy varied. This assessment aimed to identify barriers to stool adequacy in Pakistan and recommend targeted solutions to improve the AFP surveillance system. Aspects of active and passive AFP surveillance, as well as logistical considerations, were included in the assessment for 12 selected districts to obtain an understanding of barriers and potential solutions.

## 2 Materials and methods

The specific objectives of this cross-sectional operational assessment were to characterize barriers to stool adequacy, determine compliance with and quality of active surveillance visits, and make specific recommendations for improvements to Pakistan's AFP surveillance system. Pakistan's NEOC Surveillance team designed and implemented the study in collaboration with the United States Centers for Disease Control and Prevention (CDC). This activity was reviewed by CDC, deemed not research, and was conducted consistent with applicable federal law and CDC policy. As the subject of the assessment was a surveillance system, this activity was not human subjects research and informed consent was not required or obtained.

The targeted, non-random selection of districts was based on the following criteria: stool adequacy not meeting the 80% target in 2023, a decline in stool adequacy percentages from 2019 to 2023, or inconsistent stool adequacy with some years having stool adequacy rates below 80%. The districts selected were: Dera Bugti, Killa Abdullah, Noshki, and Usta Muhammad in Balochistan province; Bannu, North and Lower South Waziristan, Orakzai, and Peshawar in Khyber Pakhtunkhwa province; Gujrat, Lahore, and Rawalpindi in Punjab province; Karachi Korangi in Sindh province; and Islamabad Capital Territory. Samples were considered inadequate if any of the following conditions were not fulfilled: two stool samples were collected at least 24 h apart and both within 14 days of paralysis onset; both stool samples arrived at the national polio laboratory in good condition (i.e., adequate volume, no leaking, no desiccation, and reverse cold chain maintained) (8).

The assessment consisted of three components: a review of case investigation forms and documentation of AFP cases focusing on cases with inadequate samples with onset of paralysis during January 1, 2023–March 14, 2024, visits to active surveillance sites, and interviews with a district surveillance officer or other designated surveillance staff person assigned to selected districts and with national laboratory personnel.

The desk review of AFP cases aimed to include 20 AFP cases with inadequate stool samples and onset of paralysis during the study period from each district. In districts in which 20 or fewer AFP cases had inadequate stool samples during the study period, all cases were included. In districts with more than 20 AFP cases with inadequate samples during the study period, the three most recent cases were automatically included, and the 17 additional cases were chosen using a random number generator. In addition, data from the three most recent AFP cases with adequate stool samples were included in each district to facilitate the identification of potential differences in stool sample collection or transport which may contribute to inadequacy. The review included gathering information from the AFP case investigation file on case detection, case investigation, and sample collection, storage, and transport. For the three most recent AFP cases with inadequate and adequate samples, phone interviews with case-patients or family members were also conducted to confirm information in the case investigation files and to determine where and by whom stool samples were collected. Data from case file reviews and phone conversations were recorded onto a paper data instrument. The instrument used for AFP cases with adequate samples omitted questions related to reasons for inadequacy, and the instrument used for all except the three most recent AFP cases omitted questions related to location and responsibility for sample collection. No personally identifiable information was recorded on any of the desk review instruments.

In each district, teams visited at least eight active surveillance sites per team member. In Pakistan, the priority of an active surveillance site is designated based on the average number of pediatric patients seen and, for active sites that have previously been a part of the surveillance network, whether the facility has previously failed to report AFP cases. High-priority sites should be visited weekly, medium-priority sites twice per month (fortnightly), and low-priority sites monthly. The assessment included a mix of high-, medium-, and low-priority active surveillance sites and both government and private facilities. Where possible, teams focused on health facilities in areas of the district (called “tehsils”) with the lowest stool adequacy percentages. At each facility, the team visited the facility's AFP focal person (if applicable) and those departments where AFP case-patients are likely to be under care, such as emergency, pediatric inpatient and outpatient, and physical therapy departments, among others. During the visits, teams completed three tasks. First, teams verified district surveillance staff compliance with conducting the planned “active surveillance visits” during the previous 12 months or as much of the previous 12-month period as possible if available registers did not cover the entire 12-month period. Compliance with expected active surveillance visit frequency was assessed based on whether the number of signatures of surveillance staff in the facilities' patient registries matched the predicted frequency of active site visits according to the site's schedule by priority designation (i.e., weekly, fortnightly, or monthly for high-, medium-, or low-priority sites, respectively) and the district's surveillance work plan. Whether healthcare facility staff recalled seeing active site visits that had occurred but were not documented was also considered. Visits were considered compliant if 80% of expected visits according to the site's priority designation were verified based on the available information. Next, teams reviewed facility and department registries to look for any listed signs or symptoms or other indications/provisional diagnoses of paralysis/weakness for potential AFP cases not reported as AFP (“missed”). The missed case review covered the previous 12 months or as much of the previous 12-month period as possible if available registers did not cover the entire 12-month period. Finally, teams assessed knowledge about AFP surveillance among healthcare personnel at the facility by asking health facility staff to describe procedures for collecting, storing, and transporting stool samples by reverse cold chain; whether they had received formal AFP training/sensitization within the previous 6 months; and to whom they would report an AFP case if they identified one.

To supplement the information gathered in the field, a CDC staff member interviewed the district surveillance officer or other surveillance staff member in each selected district and staff of the Polio Regional Reference Laboratory in Islamabad. All interviews followed one of two semi-structured interview guides (one tailored for surveillance staff and one specific to laboratory staff) which included open-ended questions on various topics. The surveillance staff interview guide included questions about what factors, in their opinion, contributed to stool sample inadequacy in their district. The laboratory interview guide included questions related to causes for inadequate stools from the laboratory perspective, especially those associated with the maintenance of reverse cold chain and the use of LogTags to record temperature during sample transport. Surveillance staff interviews were conducted over the phone for most districts and in-person for staff in Islamabad and Rawalpindi; laboratory staff were interviewed in-person. Interviews varied in length from approximately 45 to 90 min. The CDC interviewer took notes during the discussion but did not record the interview. All interview participants gave verbal consent, which the interviewer recorded. Interview notes were only analyzed by the interviewer to keep responses confidential.

Fieldwork was conducted over 5 days in March 2024 in most districts by a district surveillance officer from a different province. Due to their urban environment and large numbers of healthcare facilities, Lahore, Peshawar, and Rawalpindi had two district surveillance officers assigned for fieldwork, and members of the NEOC surveillance team and CDC participated in field site visits in Islamabad and Rawalpindi. Field teams collected data on standardized paper data instruments. Field teams were trained on data collection tools and reporting by the CDC and NEOC during a virtual training (webinar) session on the 1^st^ day of fieldwork. Scans or photos of completed forms were sent daily to the NEOC and CDC team members via WhatsApp.

Quantitative results were extracted from study forms into Microsoft Excel (9). Descriptive analyses were conducted in Excel. Visit compliance as calculated as a percentage of planned visits with documentation of completion as described above, and each surveillance site was considered compliant if ≥80% of planned visits were documented or not compliant if <80% of planned visits were documented. Differences in compliance was evaluated based on site priority (high, medium, or low) and by urbanicity (urban vs. rural), and two simple logistic regression models were built to determine the statistical significance of differences based on these two predictors. Additionally, the statistical significance of differences in healthcare staff knowledge of AFP by healthcare facility staff role was tested using a simple logistic regression model comparing the odds of each staff member in each category answering each knowledge question correctly. Differences in odds ratios were considered statistically significant with *p* < 0.05. Graphical depictions of results were created and logistic regression models were conducted using R 4.3.1 software (10).

The lead author compiled and summarized qualitative information from open-ended questions included in interviews of surveillance staff and the data collection forms from visits to active surveillance sites using a content analysis approach. First, paper and electronic notes from the in-person and telephonic surveillance staff member interviews were reviewed and considered to identify common themes. Similarities and differences in individuals' responses were noted, specifically in relation to differences between those working urban vs. rural districts and in districts without or with only one vs. with multiple district surveillance officers. Similarly, for laboratory staff interviews, notes from the visit to the laboratory were analyzed and relevant information specifically related to the laboratory was highlighted for inclusion in the evaluation report to the NEOC. No direct quotations were used, and all qualitative data was reported in aggregate to maintain the confidentiality of respondents. The lead author and the NEOC team discussed the results of the qualitative analysis and the interpretation of the results, and final written results were compiled with the quantitative results by the lead author and reviewed by the NEOC team.

## 3 Results

The desk review was completed for 197 AFP cases fulfilling the study inclusion criteria from 12 districts (six to 23 cases per district). A total of 133 active surveillance sites were visited in 12 districts. Due to security constraints, field teams could not travel to North Waziristan and South Lower Waziristan, and time in Orakzai district was limited compared to other districts due to security-related travel restrictions. Interviews were conducted with 16 district surveillance officers or other persons involved in active AFP surveillance and two national laboratory personnel.

### 3.1 Desk review

A total of 158 AFP cases with inadequate stool samples and 38 AFP cases with adequate samples were reviewed. For 111 (70%) cases with inadequate samples, delayed case notification caused the case to have inadequate samples. The most common cause for delayed notification, identified for 69 cases (43%), was the health facility or provider failed to report the case. Failure to report the case occurred for health facilities and providers both within and outside of the polio active surveillance network. The next most common reason was delay of healthcare seeking by the case-patient (*n* = 34, 21%). For eight (5%) cases, a combination of both factors (i.e., a delay in presentation to the first health facility AND the health facility not reporting the case) resulted in inadequate stool samples. The death of the case-patient before two stool samples were obtained or medical reasons for not producing a stool caused sample inadequacy in 20 (13%) and eight (5%) cases, respectively. Delays in investigation caused inadequacy in 11 (7%) cases with inadequate samples, and the family refused stool collection for four (3%) case-patients. Additional reasons for inadequacy are summarized in Table 1.

**Table 1:** Reasons for stool inadequacy among 158 inadequate AFP cases.

**Reason for inadequacy**	**Number of cases**	**Percent**
Health facility failed to report AFP case	69	43%
Delay in seeking healthcare	34	21%
Death prior to obtaining two samples	20	13%
Both delay in seeking healthcare and at least one health facility failed to report	8	5%
Medical reason for delaying sample collection	8	5%
Family refused stool sampling	4	3%
Cold chain breach	4	3%
Delay in conducting case investigation	4	3%
Unknown or not clear from case file	4	3%
Poor quality sample required resampling	2	1%
Family traveled outside of area	1	1%
**Total**	**158**	**100%**

In addition to the 158 cases with inadequate samples discussed above, 38 AFP cases with adequate samples were included in the desk review to identify patterns related to case detection and reporting. The reporter of the AFP case (i.e., health facility vs. community informant) did not differ substantially between cases with adequate and inadequate samples. Among the 158 cases with inadequate samples, 133 (84%) were reported by health facilities, 23 (14%) by community informants, and two (1%) were found by district surveillance officers during routine active site visits. For the 38 cases with adequate samples, 31 (82%) were reported by health facilities, seven (18%) by community informants, and none were found by district surveillance officers.

**Figure 1:**
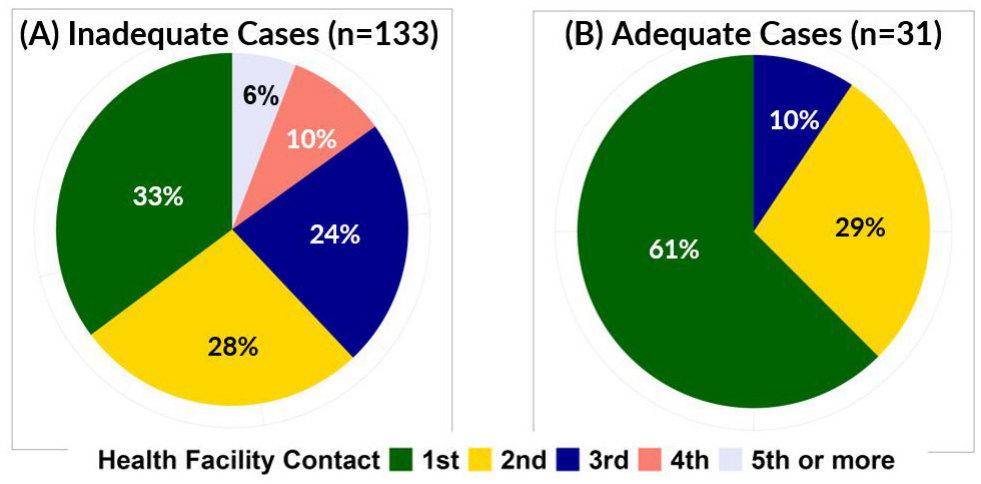
Proportion of inadequate **(A)** and adequate **(B)** AFP cases by number of health facilities visited before AFP case was reported.

The cases with inadequate samples were more likely to have been missed by one or more health facility, compared to cases with adequate samples. For the cases with inadequate samples, 60 of 158 (38%) were missed by at least one health facility that serves as an active surveillance site, compared to 4 of 38 (11%) for cases with adequate samples. Of the 164 cases reported by health facilities, 63 (38%) were reported by the first health facility or healthcare provider contacted. Specifically, 133 cases with inadequate samples were reported by health facilities, but only 44 (33%) were reported by the first health service contact, compared to 19 (61%) of the 31 cases with adequate samples reported by health facilities. All cases with adequate samples were reported by at least the third health facility where the child presented, whereas 16% of cases with inadequate samples were reported after the fourth or later health facility where the child presented (Figure 1).

### 3.2 Visits to active surveillance sites

During the review of facility registries, the field team did not identify any “missed” AFP cases (i.e., cases not included in the district's AFP line lists). Of the 133 active surveillance sites visited, 84 (63%) had documentation verifying the surveillance staff completed at least 80% of scheduled visits. Compliance with expected active surveillance visits by facility prioritization category is reported in Table 2 and by district in Table 3. Compliance varied according to the prioritization of the active site, with high-priority sites scheduled for weekly visits having the highest verifiable compliance at 78%. The medium-priority fortnightly sites and low-priority monthly sites had lower levels of compliance at 54% and 49%, respectively. Differences in visit compliance were statistically significant, with odds of visit compliance for fortnightly sites 0.32 (*p* = 0.01) and for monthly sites 0.25 (*p* = 0.002) times the odds of compliance for weekly sites. Visit compliance also varied by district, ranging from 0% in Dera Bugti and Noshki to 100% in Gujrat and Orakzai. While urban districts generally had better-documented visit compliance than rural districts, only 30% of visited active sites were considered compliant in the urban district of Karachi Korangi. Active sites in urban districts had better-documented visit compliance than rural districts (73% compared to 52%), with the exception of Karachi Korangi (30% visit compliance). The odds of visit compliance for sites in urban districts were 2.48 (*p* = 0.01) times greater than the odds of visit compliance for rural districts.

**Table 2:** Compliance with active surveillance site visits (documentation of completion of at least 80% of expected visits) by facility priority.

**Priority**	**Number in compliance**	**Total visited**	**Percent in compliance**	**Odds of compliance, compared to high-priority weekly sites (p)**
High-priority weekly sites	45	57	78%	Ref
Medium-priority fortnightly sites	19	35	54%	0.32 (0.01)
Low-priority monthly sites	20	41	49%	0.25 (<0.01)
**Total**	**84**	**133**	**63%**	

**Table 3:** Compliance with active surveillance site visits (documentation of completion of at least 80% of expected visits) by district.

**District**	**Location**	**Number in compliance**	**Total visited**	**Percent in compliance**	**Odds of being in compliance, compared to rural (p)**
Gujrat	Rural	10	10	100%	
Orakzai	Rural	4	4	100%	
Lahore	Urban	9	10	90%	
Islamabad	Urban	11	13	85%	
Rawalpindi	Urban	18	23	78%	
Peshawar	Urban	12	17	71%	
Usta Muhammad	Rural	9	13	69%	
Killa Abdullah	Rural	4	8	50%	
Bannu	Rural	4	9	44%	
Karachi Korangi	Urban	3	10	30%	
Dera Bugti	Rural	0	8	0%	
Noshki	Rural	0	8	0%	
Total	Rural	31	60	52%	2.48 (0.01)
Total	Urban	53	73	73%	Ref
**Total**	**Overall**	**84**	**133**	**63%**	

To assess healthcare workers' knowledge of AFP, 131 facility-based AFP focal persons, 195 doctors, 166 nurses, and 130 other healthcare staff were interviewed. Results are shown in Table 4. Facility-based AFP focal persons performed the best on knowledge questions, followed by doctors, who demonstrated less knowledge compared to AFP focal persons on questions related to collection and transport of stool samples and to whom AFP cases should be reported (*p* < 0.01 for each question). Both nurses and support staff performed significantly more poorly than AFP focal persons on all questions. Odds of correctly responding to questions and the statistical significance of differences in odds by category are presented in Table 4. Across all categories, health facility staff reporting attending formal training on AFP surveillance within the previous 6 months was low, ranging from 38% among support staff to 65% among AFP focal persons. Healthcare facility staff knowledge also varied by district (Supplementary Table 1). In Bannu district, all healthcare facility staff interviewed answered all questions correctly. Healthcare facility staff in Islamabad and Orakzai also scored well across multiple professional groups. However, fewer healthcare facility staff in Dera Bugti and Noshki districts were able to answer questions correctly.

**Table 4:** Knowledge of AFP surveillance by health facility staff by role.

**Question**	**Role**	**Number responding correctly**	**Total interviewed**	**Percent responding correctly**	**Odds of responding correctly, compared to AFP focal person (p)**
Define AFP	AFP focal person	124	132	94%	Ref
	Doctor	182	195	93%	0.90 (0.83)
	Nurse	131	166	79%	0.24 (<0.01)
	Support staff	75	130	58%	0.09 (<0.01)
How are stool samples collected?	AFP focal person	123	132	93%	Ref
	Doctor	155	195	79%	0.28 (<0.01)
	Nurse	98	166	59%	0.11 (<0.01)
	Support staff	55	130	42%	0.05 (<0.01)
How are stool samples stored and transported (reverse cold chain)?	AFP focal person	111	132	84%	Ref
	Doctor	120	195	62%	0.30 (<0.01)
	Nurse	73	166	44%	0.15 (<0.01)
	Support staff	48	130	37%	0.11 (<0.01)
Have you had a formal AFP training in the past 6 months?	AFP focal person	86	132	65%	Ref
	Doctor	115	195	59%	0.77 (0.26)
	Nurse	81	166	48%	0.51 (<0.01)
	Support staff	49	130	38%	0.32 (<0.01)
To whom would you report an AFP case?	AFP focal person	127	132	96%	Ref
	Doctor	165	195	85%	0.22 (<0.01)
	Nurse	128	166	77%	0.13 (<0.01)
	Support staff	97	130	75%	0.12 (<0.01)

### 3.3 Interviews with surveillance and laboratory personnel

Eighteen individuals were interviewed: 16 surveillance staff from 14 districts and two individuals from the national laboratory. Surveillance staff reported health-seeking behavior of the population and missed cases by healthcare providers outside of the AFP surveillance network as the most common reasons for stool inadequacy in their districts. In general, surveillance staff assigned to urban areas such as Lahore, Peshawar, and Rawalpindi thought that the active surveillance network may not be sufficiently large to capture all AFP cases quickly because there are too many providers and clinics to include them all in the active network and healthcare providers outside of the network are less likely to report AFP cases. In urban areas, surveillance staff also considered health-seeking behavior as an issue, as parents might choose informal or unlicensed providers because they are more accessible, have shorter wait times, may be located closer to homes than public facilities, and are cheaper than private facilities. Several surveillance staff members reported establishing rapport and maintaining close relationships with healthcare staff at their assigned facilities was key to ensuring facilities report AFP cases consistently and quickly. Surveillance staff who conduct active surveillance visits at large tertiary or teaching hospitals, which are usually found in urban locations, indicated that frequent turnover of healthcare staff and rotations of students and residents make ensuring that all healthcare staff are oriented to AFP surveillance challenging, despite reporting investing substantial time and effort in discussing AFP surveillance with healthcare staff.

In more rural areas such as Dera Bugti, Gujrat, and Killa Abdullah, surveillance staff reported high levels of poverty in their population, with families unable to afford travel with their child to a location where formal healthcare is provided. The difficulty of accessing formal healthcare pushes parents toward informal or faith-based healers, leading to delayed case notification. Some surveillance staff reported faith healers advising parents to keep their children at home while they are sick, further delaying presentation to a formal healthcare provider. Finally, in some rural areas, surveillance staff reported parents were unwilling to take children with AFP to a formal healthcare provider because of the stigma associated with being considered a polio suspect, or (rarely) because they do not want the government to have their contact information if their child is investigated for polio.

Two national laboratory staff members participated in interviews. The laboratory staff reported more samples arrived with evidence of a cold chain breach since the introduction of LogTags in mid-2023 than before the use of LogTags because of the constant recording of temperatures. However, a quantitative review of the proportion of cases requiring resampling due to cold chain breaches before vs. after the introduction of LogTags was not conducted at this time, as this topic will be reviewed once 1 year of LogTag data is available. Despite this, laboratory staff had more confidence in the results of stool sample testing due to the use of LogTags. Laboratory staff also reported most cold chain breaches recorded by LogTags seemed related to extended transit times during shipping and were observed more frequently in samples from rural districts or districts in areas with less rapid shipping networks, such as Balochistan or northern Khyber Pakhtunkhwa. Laboratory staff also reported potential seasonality with more cold chain breaches occurring during the summer months. However, this could not be confirmed because <1 year of LogTag data was available at the time of the assessment.

## 4 Discussion

The stool sample adequacy percentage remains one of the two primary performance indicators of AFP surveillance system functionality globally and in Pakistan. Even with a high non-polio AFP rate indicating a sensitive AFP surveillance system, if adequate stool samples are not obtained and tested for these cases, actual poliomyelitis cases may be missed. This assessment characterized and quantified the most common barriers to achieving high stool adequacy in key districts in Pakistan. Delays in case notification after paralysis onset were the reason for stool inadequacy in the majority (70%) of cases with inadequate samples. Notification delays have previously been identified as a barrier to robust AFP surveillance system functioning (11). The most frequent cause of delayed case notification was failed reporting by the first health facility or healthcare provider that the caretakers of the AFP case-patient visited. While most of the healthcare providers who failed to report AFP cases were outside the active surveillance site network, a substantial proportion (38%) of cases with inadequate samples included in the desk review were missed by at least one provider or facility in the active surveillance network. Additionally, only 25% of cases with inadequate samples were reported by the first healthcare contact where the child presented, compared to 61% for cases with adequate samples. This further highlights the importance of rapid recognition and reporting of AFP cases by healthcare providers.

While the visits to healthcare facilities in the active surveillance network showed the active surveillance system is generally functional and no missed AFP cases were identified during the review, only 63% of active sites documented at least 80% of expected visits occurred. Decreased compliance with active surveillance visits may contribute to delays in case notification by these health facilities. Some AFP cases could be identified by the surveillance officer during active surveillance visits, rather than reported by staff at the health facility. In these situations, decreased compliance in achieving planned active site visits could substantially lengthen the time between when an AFP case presents to a healthcare facility and case notification. Further, decreased active surveillance visit compliance may affect the likelihood of healthcare staff recognizing and reporting suspected AFP cases because fewer interactions between surveillance staff and healthcare staff leads to decreased healthcare staff awareness and sensitization about the importance of reporting AFP cases. Additionally, although several surveillance officers reported forming relationships with health facility staff and orienting them frequently to AFP is time-consuming, they also reported that these efforts pay off because maintaining strong relationships with healthcare staff ensures rapid and frequent AFP case reporting. Ensuring compliance with expected active surveillance visit frequency and maintaining a schedule of routine visits to these sites would help build and maintain these relationships between facility staff and surveillance officers.

Frequent staff turnover is a barrier to healthcare staff knowledge for large tertiary and teaching hospitals. While surveillance officers reported spending time orienting AFP focal persons and health facility staff members, rotations of junior healthcare staff and students every 3 months and frequent job changes by healthcare staff who are growing their careers make it difficult to ensure every staff member is trained in AFP identification and reporting. Results also showed differences in healthcare staff knowledge by profession, with doctors performing better than nurses or support staff. These issues could be addressed by regular training and orientations. When a new group of students or residents begin a rotation in a key department, part of their orientation to the department should include a brief refresher on recognizing and reporting AFP. Further, the team recommends all health facility staff participate in formal AFP trainings and receive frequent reminders about AFP case identification and reporting during active site visits to ensure AFP cases are recognized quickly.

Reaching healthcare providers outside of the active site network may be more challenging. Traditional or faith healers may be more common in rural areas than formal healthcare providers. Pakistan has tried to address this issue by creating a network of oriented community informants and conducting outreach to faith healers. This strategy has been somewhat successful in other countries with security-related challenges (12–14), and indeed 15% of the cases reviewed in this assessment were reported by a community informant. It is recommended the Pakistani NEOC develop a messaging plan designed to reach healthcare facilities, both formal and informal, tailored to the local area's needs. Messaging should include how to recognize AFP and to whom cases should be reported, but the message itself could be adjusted to match the health literacy level of the most common type of provider in the area. For example, using lay terms to describe AFP may be necessary in rural areas to ensure unlicensed or informal providers understand. In contrast, messages in cities could use more specific terms geared toward licensed healthcare providers outside the AFP surveillance network.

Delayed presentation to a healthcare facility was the second most common cause of delayed notification leading to stool inadequacy. While reasons for delays in seeking healthcare were not investigated during the desk review of inadequate AFP cases, surveillance staff suggested possible reasons that varied by district and urban vs. rural settings. In cities, surveillance staff attributed challenges of caregivers accessing health facilities within the polio surveillance network to the abundance of healthcare options, many of which are not a part of the AFP surveillance network. In rural areas, poverty and lack of infrastructure make visiting formal healthcare facilities, which tend to be in towns, even more difficult. These concerns echo those voiced by front-line healthcare workers in Pakistan during recent brainstorming sessions focused on identifying novel approaches for polio eradication (15). Cultural considerations, stigma, fear of confidentiality breaches, and preference for faith-based healers were mentioned, but surveillance staff found these less impactful than physical barriers to access. As part of the comprehensive communications strategy discussed above, a messaging plan to reach the public, specifically parents of children under age 15, is also warranted. Messages tailored for parents should include quickly bringing a child with sudden weakness to a formal healthcare facility.

While interview participants, including national laboratory personnel and national-level surveillance staff members, cited problems with sample storage or transport in the cold chain as a potential reason for stool inadequacy due to the need to resample, this issue only affected five cases included in this review, one of which was adequate. To follow up on these concerns, however, a detailed analysis of LogTag data should be conducted to assess seasonal trends in cold chain breaches and to identify districts most likely to have cold chain breaches. Interventions which could be implemented during hot weather or in districts with frequent breaches include adding an extra ice pack to carriers and ensuring that all ice packs are frozen solid. All areas of Pakistan should also ensure availability of alternate or backup electricity sources for cold chain and ice pack freezers.

This assessment had some limitations. First, this assessment was conducted for programmatic reasons. No power analysis was conducted to determine the sample size of facilities visited. Secondly, attributing delays in notification to health-seeking behavior vs. missed reporting was complicated by an oversight on the data collection forms used in the desk review. The desk review data collection form did not ask for the date the facility was visited until this was revised on the 3^rd^ day of the 5-day fieldwork period. Thus, this information on date of facility visit was available for only 38 of 196 cases reviewed, which may have led to incorrect attribution of delays in case reporting to the failure of the facility to report the case, when in fact, the case-patient may have presented after the 14-day window and would have been inadequate regardless of whether the first healthcare contact reported it. To address this limitation, the team recommends the NEOC ensure the names and dates of visited healthcare facilities are included in the electronic surveillance database and analyses are conducted routinely to identify active sites that are repeatedly missing AFP cases and areas where delays in seeking healthcare contribute substantially to stool inadequacy. The interventions recommended above could then be targeted to districts where they will have the most impact. While the purpose of the study was to identify barriers to stool adequacy from the polio eradication program perspective, additional barriers to stool adequacy such as public perception of polio risk, social and cultural norms, and parent perception of healthcare quality were not assessed but could contribute to low or decreasing stool adequacy in Pakistan.

In conclusion, this assessment reinforces the strengths of Pakistan's AFP surveillance system, as little evidence of unreported AFP cases was found. Although Pakistan has one of the most robust AFP surveillance systems globally, continual improvement is crucial for achieving eradication of wild poliovirus in the last reservoirs in the world. Understanding the most frequent barriers to stool adequacy is an important first step in designing tailored approaches to address and mitigate them and ensure that all poliomyelitis cases are detected.

## Data Availability

The original contributions presented in the study are included in the article/Supplementary material, further inquiries can be directed to the corresponding authors.
